# Supplementary figures and images for: Soil bacterial biodiversity is driven by long-term pasture management, poultry litter, and cattle manure inputs
Source: PeerJ. 2019 Oct 1;7:e7839. doi: 10.7717/peerj.7839 (PMC6777480; doi:10.7717/peerj.7839)

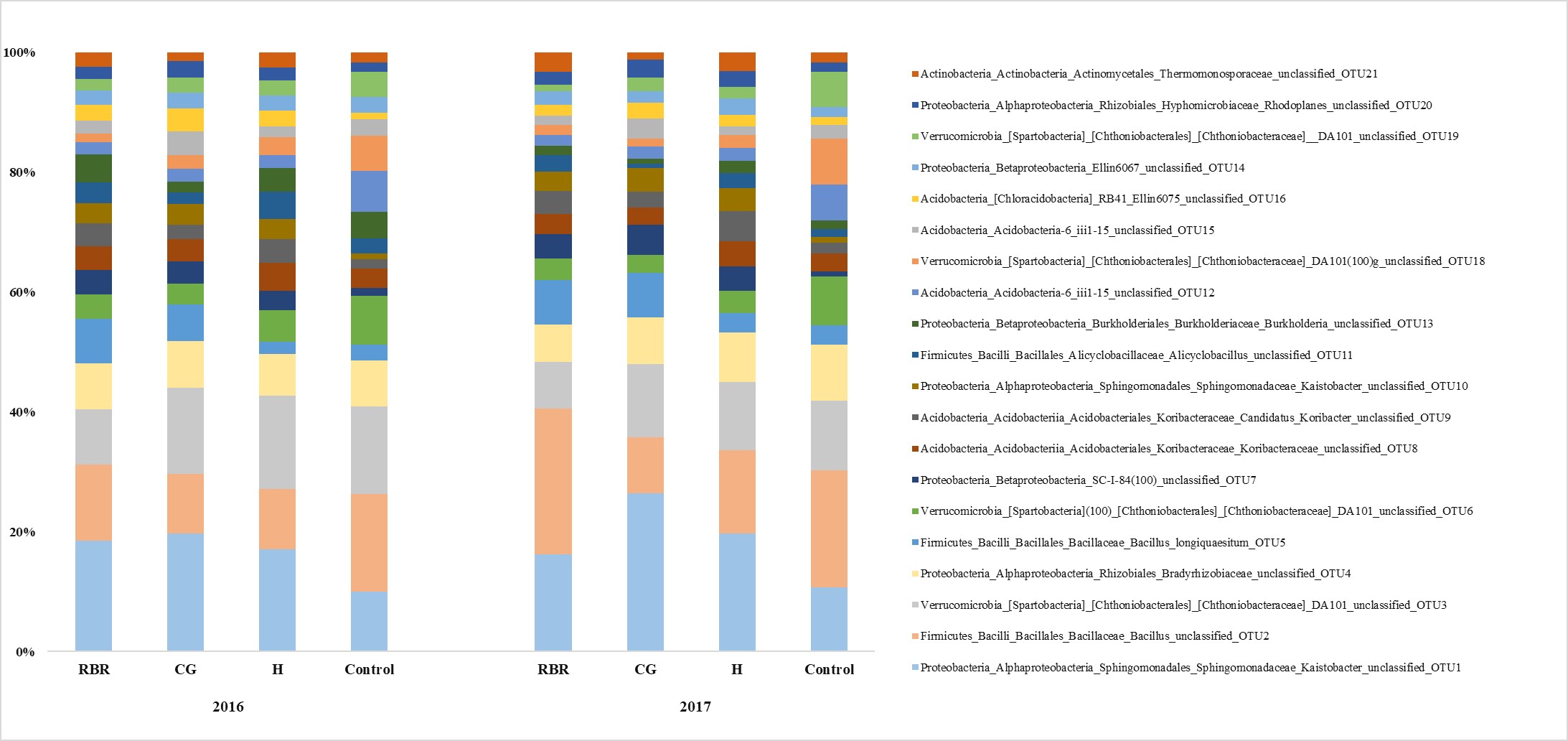

Supplement: Figure S1 [file peerj-07-7839-s001.jpg]

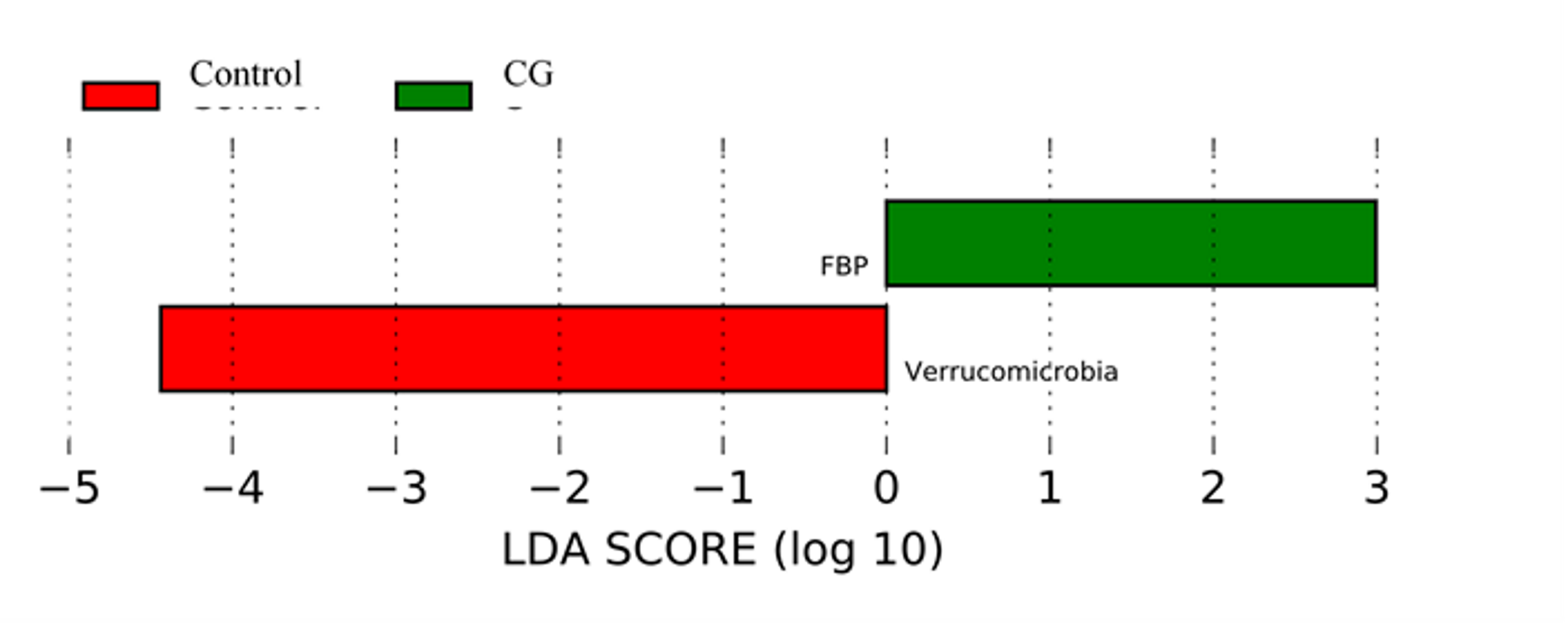

Supplement: Figure S2 — Linear Discriminant Analysis Effect Size (LefSe) analysis showing abundance of Verrucomicrobia and FBP phyla were significantly different between the control and the continuously grazed (CG) pasture management treatments. [file peerj-07-7839-s002.png]
